# Immuno-Oncotherapeutic Approaches in Advanced Hepatocellular Carcinoma

**DOI:** 10.3390/vaccines8030447

**Published:** 2020-08-08

**Authors:** Robin Park, Fariha Eshrat, Mohammed Al-Jumayli, Azhar Saeed, Anwaar Saeed

**Affiliations:** 1 MetroWest Medical Center, Tufts University School of Medicine, Framingham, MA 01702, USA; robin.park@mwmc.com; 2 Department of Medicine, Division of Medical Oncology, Kansas University Cancer Center, Kansas City, KS 66160, USA; feshrat@alfaisal.edu (F.E.); mjumayli@gmail.com (M.A.-J.); 3 Department of Pathology and Laboratory Medicine, Kansas University Medical Center, Kansas City, KS 66160, USA; asaeed2@kumc.edu

**Keywords:** hepatocellular carcinoma, PD-L1, CTLA-4, oncolytic viruses, antigen peptide vaccines, DC vaccines, cytokine induced killer cells, CAR T cells

## Abstract

Advanced hepatocellular carcinoma has limited treatment options, but there has been extensive growth recently with cabozantinib, regorafenib, lenvatinib, nivolumab, atezolizumab, and bevacizumab, which are some of the treatments that have received FDA approval just over the last three years. Because HCC tumor microenvironment is potentially immunogenic and typically characterized by inflammation, immunotherapy has been proposed as a potential novel therapeutic approach, which has prompted studies in advanced HCC patients investigating various immune-therapeutic strategies such as CAR-T cell therapy, checkpoint inhibitors, and onco-vaccines. The anti-PD-1 checkpoint inhibitors nivolumab and pembrolizumab have been FDA approved as a second line treatment in patients who progressed or are intolerant to Sorafenib. To build up on the success of PD-1 monotherapy, combinatorial regimens with PD-1/PD-L1 inhibitors plus VEGF targeted agents have shown positive results in various malignancies including HCC. The combination of atezolizumab plus bevacizumab is the new addition to the HCC treatment armamentarium following a pivotal study that demonstrated an improvement in OS over frontline sorafenib. Other novel immune-based approaches and oncolytic viruses are in the early phases of clinical evaluation. These innovative approaches enhance the intensity of cancer-directed immune responses and will potentially impact the outlook of this aggressive disease.

## 1. Introduction

As the most common primary hepatic malignant tumor, hepatocellular carcinoma (HCC) is the fourth leading cause of cancer-related mortality in the United States [1]. Because of the increasing incidence of HCC, research into HCC management has become more crucial. The average survival of untreated patients is only 9 months, and most patients present with advanced stages of HCC and significantly diminished hepatic function at the time of diagnosis. Despite variance in staging systems, variables that are taken into account in defining advanced HCC include the presence of portal vein invasion and extrahepatic metastatic disease in addition to preserved functional status and liver function. The trials described herein in general conform to this definition, although they vary in whether patients were treatment naïve or experienced with prior systemic therapy. Therefore, advances in immune therapeutics are crucial for developing a more efficacious option for adjunct therapy in HCC management. The approval of sorafenib in the systemic management of HCC in 2007 was groundbreaking. Since then, the Food and Drug Administration (FDA) has approved numerous therapies with effective systemic results including immunotherapeutic options as is discussed below.

Immune checkpoint inhibitors have recently been approved for use in advanced stage settings as single agents as well as in combination therapy. HCC can mount a strong immunologic response because it is highly vascular, contains numerous mutations, and exhibits several specific cell surface antigens [2,3,4]. This makes immunotherapeutic agents an appealing treatment modality with a relatively low toxicity profile.

The mechanisms of primary resistance to immunotherapeutic approaches in HCC may be explained by the following: in the natural state, T-cell response is elicited by the binding of T-cell receptors (TCRs) to the major histocompatibility complex (MHC) on antigen presenting cells (APCs) and the expression of co-stimulatory molecules on both cells. As malignant cells have reduced expression of MHC-I, antigens are not being naturally developed and presented [5]. This prevents activation of T cells and inhibits a robust T-cell response. Another mechanism by which hepatic cells and hence, HCC cells can cause immunosuppression is through exhibiting a lower number of co-stimulatory particles like B7-1 or B7-2, which is expected to result in T cell anergy and leads to immune escape [6,7].

Although they have been meticulously researched for HCC therapy, targeted vaccines that result in efficacious cytokine release or non-specific T-cell activation are yet to be discovered. Nonetheless, multiple alternative immune-based treatments have shown pronounced improvement of advanced HCC. Additionally, these treatments also have lower toxicity profiles.

**Figure 1:**
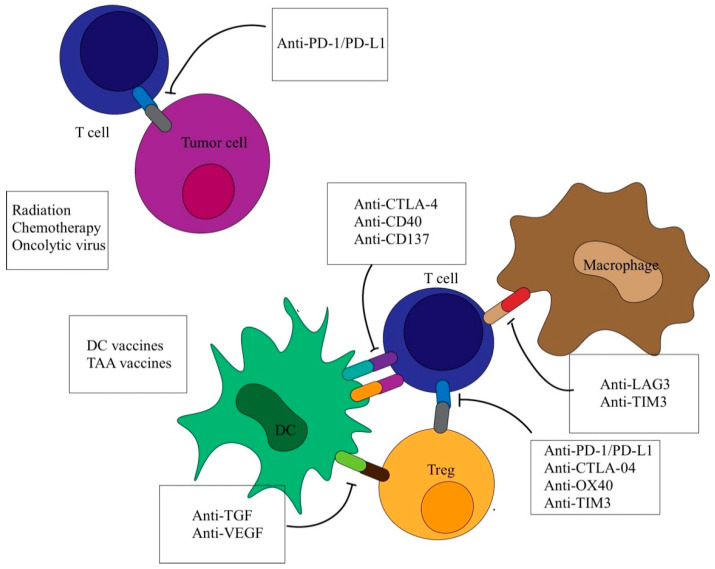
Immunotherapeutic mechanisms. Immunotherapeutic mechanisms. Immune therapy targets the interface of intercellular interaction among immune cells and between immune cells and tumor cells. Such treatment modalities inhibit well-known immunological checkpoints such as the PD-1/PD-L1 and B7/CTLA-4 axes to stimulate anti-tumor immune responses. Other immune therapeutic modalities engage the antigen presentation mechanism in order to achieve the same end, for example, by cancer vaccination. Still other treatment modalities, including oncolytic therapy, target the direct destruction of tumor cells. Abbreviations: CTLA-4, cytotoxic-T lymphocyte antigen-4; PD-1/PD-L1, programmed death-1/programmed death ligand-1; LAG-3, lymphocyte activation gene 3; TIM-3, T-cell immunoglobulin-3; TGF, transforming growth factor; VEGF, vascular endothelial growth factor.

Studies have shown HCC cells with overexpression of programmed death-1 (PD-1) can elicit weaker immune reactions by suppressing T-cell response and resulting in uncontrolled cell division [8,9]. Amplified MHC class II molecule expression in hepatocellular carcinoma result in T-cell anergy as co-stimulatory molecules cannot be identified by other antigen-presenting cells. Further research into the interactions of HCC with host immune systems can reveal invaluable data for improving immunotherapy for HCC management [10] (Table 1) (Figure 1).

In this article, we describe the previous clinical trials that were pivotal in the development of direct and indirect immunotherapeutic approaches in advanced HCC and introduce important ongoing clinical trials.

## 2. Indirect Immunotherapy: Immune Checkpoint Inhibitors

Checkpoint inhibitors have become a vital pillar of modern immunotherapy. The most extensively analyzed targets include: programmed death ligand-1 (PD-L1), PD-1, OX-40, lymphocyte activation gene 3 protein (LAG-3), T-cell immunoglobulin and mucin-domain-containing (TIM-3), cytotoxic T lymphocyte-associated antigen-4 (CTLA-4), V-domain Ig suppressor of T cell activation (VISTA), and B and T lymphocyte attenuator (BTLA) [11,12]. Among them, PD-1/PD-L1 and CTLA-4 have shown to be most successful in HCC as is discussed below.

### 2.1. PD-1/PD-L1 Inhibitors

PD-1/PD-L1 inhibitors such as pidilizumab, pembrolizumab, and nivolumab may potentially halt malignant cell growth and activate the autologous T-cell dependent destruction of cancer cells.

Studies in patients with cirrhosis and concurrent hepatocellular carcinoma show increased PD-1 expression on cytotoxic T-cells during the effector phase of humoral immune response [13]. As we know that hepatic resection is associated with a rise in PD-1 expression on cytotoxic T-cells, the destruction of healthy hepatic tissue by HCC growth is thought to cause the increased expression of PD-1 on cytotoxic T-cells in HCC patients. This in turn results in a faster progression of hepatocellular carcinoma [14]. Hence, anti- PD-1 and anti-PD-L1 therapies are an exciting new target for HCC management.

Nivolumab monotherapy was assessed as part of the CheckMate-040 phase I/II trial [15]. This study applied a strict inclusion and exclusion criteria for patient enrollment which included the following: (1) intermediate or advanced stage of HCC; (2) healthy hepatic function as defined by Child-Turcotte-Pugh class of A or better; and (3) eligibility for systemic therapy management including demonstration of disease progression or intolerance to sorafenib [16]. Primary endpoints were safety and objective response rate (ORR). Dose escalation recorded 15% tumor response while dose-expansion showed a 20% ORR. Within a mean timeframe of 17 months, patients showed a robust response to the treatment, with the disease progression stabilizing in up to 45% of patients over six months. Objective tumor responses were consistent across HCC population risk factors, and no significant differences were noted when comparing patients who had taken sorafenib as part of their treatment regimen to those who had never taken sorafenib. High grade treatment-related adverse events (TRAEs) were seen in 20% of all patients on nivolumab. No treatment-related deaths were recorded but up to 3% of the patients were forced to discontinue the drug. Although only 5% of patients reported high-grade immunologic hepatitis that necessitated the use of systemic steroids, serum transaminases were raised in up to 20% of the patients.

Nivolumab was granted accelerated approval by the FDA for its promising efficacy and acceptable toxicity profile. Nivolumab is now a viable second line option for the management of HCC, after sorafenib. An active phase III trial named CheckMate-459 is currently evaluating nivolumab versus sorafenib as the first-line management through a randomized study. The interim results of this study were published in the 2019 European Society of Medical Oncology (ESMO) meeting. Primary endpoint was overall survival (OS). Results showed a favorable ORR of nivolumab over sorafenib (ORR, 15 vs. 7%, nivolumab vs. sorafenib); however, the primary endpoint of OS did not reach statistical significance (HR, 0.85, 95% CI, 0.72–1.02). The decision to enroll a population not selected for PD-L1 has been cited as the major reason for this failure to meet the primary endpoint. Supporting this notion is that ORR in the PD-L1 >1% population was significantly more favorable for nivolumab therapy (ORR, 28 vs. 9%, nivolumab vs. sorafenib). Though the PD-L1 >1% population made a small minority of patients in the CheckMate-040 trial (18%), PD-L1 may be useful for selecting a responder population [17].

KEYNOTE-224 is a phase I/II trial that reviewed the use of pembrolizumab in the management of advanced HCC with continued disease progression despite the use of sorafenib. Primary end point was ORR. The study randomized 104 patients in total. Pembrolizumab therapy demonstrated an ORR of 16.3%, an impressive median progression-free survival (mPFS) of 4.8 months and a durable response of 6 months or better in 94% of patients. The updated interim results showed a mPFS of 4.9 months (95% CI, 3.5–6.7) and median OS (mOS) of 13.2 months (95% CI, 9.7–15.3) [18]. No new adverse events of pembrolizumab therapy were noted, and the treatment demonstrated no increased risk of viral infections.

Based on the positive results of the KEYNOTE-224, a phase III trial was initiated to compare head to head with pembrolizumab and best supportive care in previously treated advanced HCC patients. The preliminary results of the KEYNOTE-240 were recently presented after a median follow-up of 13.8 months and randomization of 413 patients (278 to pembrolizumab vs. 135 to placebo). The primary endpoints were OS and PFS. The results show improved OS (HR, 0.78; one sided *p* = 0.0238) and PFS (HR, 0.78; one sided *p* = 0.0209) for pembrolizumab versus placebo. ORR was 16.9% (95% CI 12.7–21.8%) for pembrolizumab vs. 2.2% (95% CI, 0.5–6.4%) for placebo. Pembrolizumab also showed a durable response (median DOR: 13.8 months [1.5–23.6+]). The survival advantage for pembrolizumab as oppose to placebo did not reach significance per the prespecified statistical plan. This is possibly related to the impact of subsequent therapies received by the placebo arm patients including checkpoint inhibitors. Detailed analysis of the type of post-trial therapies received by both groups and the associated PFS in each will largely help us explain the ameliorated survival benefit seen in this trial. Adverse effect profile in keynote-240 was consistent with that of established pembrolizumab monotherapy [19].

So far, there have been no trials evaluating anti-PD-L1 agents as monotherapies in advanced HCC. However, these agents have produced positive results in combination therapy trials as explained in a later section.

### 2.2. CTLA-4 Inhibitors

CTLA-4 is a competitive inhibitor of the costimulatory molecule B7-1 and B7-2 with high affinity for CD28. By preventing B7 and CD28 ligation, CTLA-4 suppresses signal 2 in the antigen presenting cell—T cell interaction thereby leading to T-cell suppression; in this setting, anti-CTLA-4 releases this checkpoint, resulting in enhanced anti-tumor immunity [20]. To date ipilimumab and tremelimumab are the two anti-CTLA-4 agents approved for use in advanced cancer and among them, tremelimumab was the first immune checkpoint inhibitor to be tested in HCC [21]. Another open-label study of 19 patients showed similar results (ORR 26%, median time to response (mTTP) 7.4 mo, mOS 12.3 mo) [22]. More recently, the results of Study 22 (NCT02519348) which evaluated durvalumab, tremelimumab, and the combination of both were presented at the American Society of Clinical Oncology (ASCO) 2020 Virtual Meeting. In this multi-arm phase II study in which safety was the primary endpoint patients who were immune checkpoint inhibitor-naive, and were intolerant to or refused sorafenib were randomized to one of four arms and in total 69 patients were randomized to the tremelimumab arm. Results showed favorable efficacy for tremelimumab (mOS, 17.1 mo, 95% CI, 10.9-NR; ORR, 7.2%, 95% CI, 2.4–16.1; median duration of response (mDOR) 24.0 mo). Adverse events were comparable to previously reported rates for tremelimumab (grade 3–4 TRAEs 42.0%) [23].

Although ipilimumab has not been evaluated as a single agent in HCC, it was recently approved for use in combination with nivolumab.

### 2.3. Combination Therapy with Immune Checkpoint Inhibitors

Rational treatment combinations of immune checkpoint inhibitors are emerging as one of the most effective ways to overcome primary resistance to immune checkpoint inhibitor monotherapy in advanced cancer. This strategy has demonstrated excellent efficacy and tolerable toxicity in other types of cancer including non-small cell lung cancer, melanoma, and renal cell cancer and are already part of the therapeutic armamentarium in the treatment of these tumors in the advanced stages [24,25,26]. Furthermore, following the approval of sorafenib in 2008 and until recently with the approval of targeted and immunotherapeutic agents, many monotherapy agents of various mechanisms of action failed to yield positive results in multiple clinical trials [27,28,29,30,31]. Likewise in HCC, recently published studies have demonstrated modest efficacy in certain combination regimens and paradigm-shifting results in other trials.

In the first line setting, the landmark results of the phase III IMBrave-150 trial which evaluated the combination of bevacizumab (anti-vascular endothelial growth factor (VEGF)) and atezolizumab were recently published. Evaluation of this combination was prompted by pre-clinical studies demonstrating that anti-VEGF therapies reduce VEGF associated immune suppression in the tumor microenvironment and promote the increase of tumor infiltrating lymphocytes, thereby enhancing anti-PD-1 or PD-L1 efficacy [32,33,34,35,36]. Primary endpoints were OS and PFS in the intention to treat population. This study enrolled 485 patients with systemic treatment-naive advanced stage HCC and randomized to atezolizumab plus bevacizumab or sorafenib treatment arms. Atezolizumab plus bevacizumab demonstrated prolonged OS (HR, 0.58, 95% CI, 0.42–0.79) and PFS (HR, 0.59, 95% CI, 0.47–0.76) and higher ORR (odds ratio, 2.77, 95% CI, 1.62–4.74) as well as comparable toxicity (grade 3–4 TRAEs, 56.5 vs. 55.1%, atezolizumab plus bevacizumab vs. sorafenib). With this trial, this novel combination therapy became the first therapeutic regimen to induce prolonged survival over sorafenib and resulted in the FDA approval for use in the first line setting [37].

Furthermore, the combination of durvalumab plus ipilimumab has shown promising potential efficacy. In this aforementioned study (NCT02519348), two of the four randomized arms were the combination regimen arms of durvalumab plus tremelimumab with different dosing (75 patients, tremelimumab 300 mg plus durvalumab 1500 mg; 84 patients, tremelimumab 75 mg plus durvalumab 1500 mg). Results showed promising survival (mOS 18.7 mo, 95% CI, 10.8-NR) and excellent tumor responses (ORR, 22.7, 95% CI, 13.8–33.8%) in the T300 + D arm [23]. Of note, the T300 + D treatment regimen is currently being evaluated in the phase III HIMALAYA study (NCT03298451) in the first line setting.

In the second line or later settings, nivolumab plus ipilimumab has been evaluated in the CheckMate-040 trial. In this phase I/II study, both sorafenib naive and experienced patients were enrolled and 148 patients were randomized to three nivolumab plus ipilimumab arms with different dosing. Patients in the nivolumab 1 mg/kg plus ipilimumab 3 mg/kg arm showed favorable ORR (32%) and overall survival (mOS, 23 mo, 95% CI, 9-NR). Overall, toxicity was acceptable (grade 3–4 TRAE, 37%). Based on this study, nivolumab plus ipilimumab was approved for use in the second line setting in addition to nivolumab monotherapy [38,39].

In addition, there are several ongoing clinical trials testing other novel combinations in advanced HCC in first line as well as second line or later settings (Table 2).

## 3. Indirect Immunotherapy: Cancer Vaccines

In recent years, vaccines that can be modified to target antigens on tumor cells have been developed. These vaccines activate the autologous cellular and humoral immunity for strong immunogenic reactions. Thus, tumor cells can be phagocytosed and cleared through the use of the numerous specific immunologic triggers mentioned below.

### 3.1. Antigen Peptide Vaccines

Studies have identified many protein antigens that serve as outstanding vaccine targets for the management of hepatocellular carcinoma. The most prominent of these include glypican 3 (GPC3), alpha-fetoprotein (AFP), NY-ESO-1, SSX-2, human telomerase reverse transcriptase (hTERT), hepatocellular carcinoma-associated antigen-587 (HCA587), and melanoma antigen gene-A (MAGE-A).

As AFP is produced by fetal hepatic cells, it is present during the early stages of differentiation of the immune system. Therefore, despite the highly specific and pronounced expression of AFP in hepatocellular carcinoma cells, the immunologic reaction to AFP is insufficient as an acquired immune tolerance has developed. However, studies have shown that using recombinant AFP from rats can produce a more significant immune reaction through cross-reactivity between the newly introduced and autologous cells [40].

Furthermore, phase I studies conducted with different peptide vaccines have shown encouraging results [41,42]. A recently conducted, phase II study of a vaccine for hepatocellular carcinoma directed toward GPC3 molecules reflected the potency of this treatment modality. Although the study followed only a small number of patients, the patients who received ten doses of the vaccination post-operatively for 12 months had a significantly lower risk of HCC recurrence than their unvaccinated counterparts. A recurrence rate of 24% was seen in the group who received a combination therapy of surgery and vaccination while the recurrence rate was twice as high in the group that only received surgery after 1 year (*p* = 0.047). Furthermore, the recurrence rates were 52.4% versus 61.9% at 2 years (*p* = 0.387) [43]. This marks a significant stride toward the advancement of vaccine immunotherapy for HCC treatment.

### 3.2. Dendritic Cells (DC) Vaccines

As the most important antigen-presenting cell, dendritic cells (DCs) play a vital role in activating the primary immune response and inducing the differentiation of T lymphocytes. Therefore, DCs have been the target of recent research into immune cell-mediated vaccine modalities. The following phase I/II trial assessed the effect of DC vaccines on the OS of patients with advanced HCC. Total of 13.3% of the patients treated with autologous DCs that had been pulsed with HCC antigens showed partial radiologic improvement while 60% of them showed disease stabilization. This study used the best palliative care as their control group. As the tolerance and safety of these therapies have already been proven, the positive effect of DC vaccines on HCC prognosis makes this treatment more favorable. DC vaccines are therefore a promising option for adjuvant therapy or palliative care in advanced HCC management [44].

### 3.3. Oncolytic Virus Vaccines (OVs)

Oncolytic viruses (OVs) can bind to and target tumor cells with high specificity. As they multiply inside the cancer cells, the weakly immunogenic tumor cells allow the intracellular viruses to grow exponentially and result in cell lysis [45,46,47,48]. This releases tumor particles that can sensitize the host immune system and elicit a robust immunologic response through APCs such as DCs [49,50].

The promising potential of OVs has been repeatedly demonstrated. OVs have a low toxicity profile as the neighboring healthy cells remain unaffected and tumor cells are damaged with high specificity, these advantages have made OVs a more favorable option for adjuvant therapy. Studies have demonstrated low likelihood of contracting resistant viral infections or introducing cancer mutations into healthy cells. Treatment-resistance to OVs is less likely because they mount an antitumor response through multiple pathways. Also, the interesting pharmacokinetics of OVs allows intracellular OV dose to increase over time from viral replication while drug concentrations traditionally decrease over time in other treatments [51,52]. These favorable findings have cemented researchers’ interest in oncolytic viruses.

Some examples of genetically engineered oncolytic viruses that have shown efficacy in advanced hepatocellular carcinoma management include JX-594 and cancer-favoring oncolytic vaccinia virus (CVV). JX-594 can improve tumor-cell mediated immunogenicity. This is a result of increased β-galactosidase and granulocyte macrophage-colony stimulating factor (GM-CSF) translation in host cells because of gene inactivation of thymidine kinase by OVs. A phase II study has tested and confirmed the potential of a JX-594 oncolytic virus-based treatment of HCC [53]. A recent randomized study on animal models evaluated the effect of CVV oncolytic virus on metastatic hepatocellular carcinoma. Treatment groups that received CVV, with or without additional sorafenib, showed a significant decrease in the occurrence of metastasis when compared to groups treated solely with sorafenib [54]. Therefore, human trials of OVs would be the appropriate next step to pursue the advancement of this therapy.

## 4. Direct Immunotherapy: Adoptive Cell Therapy

Adoptive cell therapy targets malignant cells through genetic modification of autologous immune systems. Exposure to tumor antigens and/or specific cytokines activates the autologous immune cells. These cell lines can then be extracted and developed in vitro before re-injecting them into the patients. Below we explore the most extensively studied cell lines for adoptive therapy that have been efficacious in HCC management [55,56,57].

### 4.1. Chimeric Antigen Receptor T (CAR-T) Cells

Chimeric antigen receptor T cells (CAR-T) have shown exceptional therapeutic results in hematologic malignancies. Although they are MHC nonspecific, these T cells are engineered to bind to specific tumor-associated antigens (TAAs) and mount a more aggressive attack on malignant cells [58,59].

CAR-T cells are currently being developed to treat hepatocellular carcinoma and other solid malignancies [60,61]. As these malignancies often exhibit significant pleomorphism, malignant cells that do not carry the antigen CAR-T cells were engineered to target, can survive and result in cancer relapse [62,63]. However, exciting advances in the field have resulted in the recently refined fourth generation of CAR-T cells. These are transgenic “payload or TRUCK” [64]. These cells can enhance the host immune response by triggering the immune system through an increase in IL-12 production. Hence, this allows for the destruction of antigen-negative cells too.

GPC3 is the most extensively studied TAA in the production of CAR-T cells for hepatocellular carcinoma. Although this HCC marker was previously associated with poor prognosis, its specificity and overexpression in hepatoma cells have created an exciting opportunity to engineer specific CAR-T cells against GPC3-positive cell lines [65,66]. These CAR-T cells were proven efficacious ex-vivo and in mice models, with clinically significant prolonged survival [67,68]. At the same time, CAR-T cells targeting GPC3 and asialoglycoprotein receptor 1 (ASGR1) showed improved toxicity profiles and were still capable of mounting effective immunologic reactions during each ex vivo and in vivo interaction [69,70]. Hence, clinical trials studying the utility of CAR-T-based HCC therapeutic regimens would be the next step in evaluating CAR-T cell therapy for HCC (Table 2).

### 4.2. Cytokine-Induced Killer (CIK) Cells

Cytokine-induced killer cells (CIK) can activate antitumor responses through MHC independent identification of tumor markers [71,72]. A recent phase III trial found clinically significant time to recurrence (TTR) in a randomized study evaluating the radical resection of hepatocellular carcinoma along with adjuvant CIK cell therapy. Patients in the treatment group were administered four cycles of CIK therapy, while those in the control group were only observed. The median TTR was found to be 13.6 months versus 7.8 months in the group receiving CIK therapy and the control, respectively (*p* = 0.01). Toxicities reported ranged from grade 1 events to grade 2 events. However, no significant differences were noted in disease incidence, overall survival (OS), and disease-free survival (DFS) between the various treatment groups [73]. Nonetheless, a meta-analysis showed that a combination of dendritic cells, CIK cells, and transarterial chemoembolization (TACE) therapy can increase OS, disease control rate (DCR), ORR, and quality of life for the patient [74].

### 4.3. Natural Killer (NK) Cells

NK cells have long been known for their role in the natural defense against cancer cells as an important part of the immune system. Conversely, the lack of specific sensitization in a natural setting also leads to simultaneous injury of normal liver tissue during NK cell-mediated immune response to cancer cells [75]. Therefore, numerous research studies have created genetically engineered components to enhance the specificity of NK cell response to HCC. One study found that submerging HCC cells in 5 μmol/L of sorafenib over forty-eight hours renders them more susceptible to the destruction of NK cells. Other researchers have shown the potential of NKG2D, a genetically modified NK cell-activating receptor that exhibited improved NK cell cytotoxicity in ex-vivo models and mouse models. Finally, K562-mb15-41BBL is an effective ex-vivo HCC cell line that improves the efficacy of NK cells targeting hepatocellular carcinoma [76].

### 4.4. Tumor-Infiltrating Lymphocytes (TILs)

The presence of lymphocytes within the tumor microenvironment (TME) has been noted in the past and have been dubbed tumor-infiltrating lymphocytes (TILs). An extensive phase I research testing TILs on hepatocellular carcinoma found an impressively low toxicity profile for TIL therapy [77]. However, the technical obstacles involved in purifying and cultivating the TILs have posed a significant barrier in promoting their widespread use. The theoretical approach currently being studied involves extracting specialized TILs from the affected patient and growing their cell lines with the help of IL-2 and anti-CD3 antibodies before reinjecting these highly specific cells back into the patient to target and suppress tumor growth and differentiation [78,79,80,81].

## 5. Conclusions

Immune therapeutics have begun to change the landscape of systemic therapy regimens in both first line and later line settings in advanced HCC. Chiefly among them, the immune checkpoint inhibitors have been the most impactful especially in combination therapies, and also as monotherapies. The landmark results of the IMBrave150 trial as well as the ongoing trials testing combination immune checkpoint inhibitor therapies will certainly further solidify the ever-growing role of immune checkpoint inhibitors in not just HCC, but across advanced tumors in general. Despite these hopeful and promising advances, the failures of immune checkpoint inhibitor monotherapy trials to meet primary endpoints remind us the need for better patient selection and the need for biomarker development. In addition to the immune checkpoint inhibitors, the cancer vaccines and adoptive cell therapies have been studied in advanced HCC. However, although these therapeutic modalities have shown efficacy in different cancer types, such as melanoma and blood cancers, they have so far not demonstrated efficacy in HCC. Nonetheless, their strong immunological rationale suggests that such therapies may be promising companions alongside other established systemic anti-cancer therapies including the immune checkpoint inhibitors. The results of ongoing trials evaluating such rational combination therapies are much anticipated.

## Figures and Tables

**Table 1:** Main immunotherapeutic strategies for hepatocellular carcinoma (HCC).

Immunotherapeutic Strategies for HCC		
**Direct**		
ACT *	CIK cells *	CIK -valproate
		DC-CIK with TACE *
	TLS	HCC
	NK cells *	NK with K562-mb1541BBL
		Sorafenib
		NKG2D *
	CAR-T *	Targeting GPC3 *
	(Generation 1–4)	Targeting GPC3 and ASGR1 *
**Indirect**		
Oncolytic viruses	CVV, JX-594	
HCC vaccines	Cell vaccines	HCC cells with GM-CSF *
	Antigen Peptide Vaccines	AFP *, GPC3, SSX-2
	DC vaccines	Nifuroxazide
		DC-loaded TCL *
Immune checkpoint inhibitors	CTLA-4 Inhibitors *	Tremelimumab Ipilimumab
	PD-1 Inhibitors *	Nivolumab
		Pembrolizumab
		Pidilizumab
	PD-L1 inhibitors *	Atezolizumab
		Durvalumab

* Abbreviations: ACT, adoptive cell therapy; CIK, cytokine induced killer cells; DC-CIK with TACE, dendritic cell-CIK with transarterial chemoembolization; NKG2D, natural killer group 2D receptor; CTLA-4, cytotoxic-T lymphocyte antigen-4; PD-1/PD-L1, programmed death-1/programmed death ligand-1.

**Table 2:** Ongoing clinical trials evaluating immunotherapeutics in advanced HCC.

NCT Number	Status	Conditions	Interventions	Phases
PD-1/PD-L1				
NCT03419481	Recruiting	Hepatocellular Carcinoma	Pembrolizumab	Phase 2
NCT01658878	Active, not recruiting	Hepatocellular Carcinoma	Nivolumab + Sorafenib	Phase 1/2
NCT02940496	Active, not recruiting	Hepatocellular Carcinoma	Pembrolizumab in combination with various drugs	Phase 1/2
NCT03857815	Recruiting	Hepatocellular Carcinoma	Anti-PD-1 + radiation	Phase 2
NCT03914352	Recruiting	Hepatocellular Carcinoma	Anti-PD-1 + TACE	*n*/a
NCT03722875	Recruiting	Hepatocellular Carcinoma	SHR-1210 (anti-PD-1) + apatinib	*n*/a
NCT03966209	Recruiting	Hepatocellular Carcinoma	JS001(PD-1 inhibitor)	Phase 1
NCT03732547	Recruiting	Hepatocellular Carcinoma	Anti-PD-1	Phase 2
NCT03071094	Active, not recruiting	Hepatocellular Carcinoma	Nivolumab + Pexastimogene Devacirepvec (Pexa Vec)	Phase 1/2
NCT03463876	Active, not recruiting	Hepatocellular Carcinoma	SHR 1210 + apatinib	Phase 2
NCT02989922	Unknown	Hepatocellular Carcinoma, Non-Resectable	SHR-1210	Phase 2
NCT03412773	Active, not recruiting	Hepatocellular Carcinoma	BGB-A317 (Tislelizumab, anti-PD-1) + Sorafenib	Phase 3
NCT03419897	Active, not recruiting	Hepatocellular Carcinoma	BGB-A317	Phase 2
NCT03785210	Recruiting	Various solid cancers including hepatocellular carcinoma	Nivolumab in combination with various drugs	Phase 2
NCT03655613	Recruiting	Hepatocellular Carcinoma, Renal Cell Carcinoma	Nivolumab in combination with APL-501 or APL-101	Phase 1/2
NCT03605706	Recruiting	Hepatocellular Carcinoma	SHR-1210 + FOLFOX4	Phase 3
NCT02423343	Active, not recruiting	Various solid cancers including hepatocellular carcinoma	Nivolumab + Galunisertib	Phase 1/2
NCT03259867	Active, not recruiting	Various solid cancers including hepatocellular carcinoma	Anti-PD-1	Phase 2
NCT03973112	Recruiting	Hepatocellular Carcinoma	HLX10 (anti-PD-1) + HLX04	Phase 2
NCT03645980	Recruiting	Hepatocellular Carcinoma	DKN-01 + Sorafenib	Phase 1/2
NCT03222076	Recruiting	Hepatocellular Carcinoma	Nivolumab + Ipilimumab	Phase 2
NCT02702401	Active, not recruiting	Hepatocellular Carcinoma	Pembrolizumab	Phase 3
NCT03316872	Recruiting	Hepatocellular Carcinoma	Pembrolizumab + radiation	Phase 2
NCT02886897	Recruiting	Various solid cancers including hepatocellular carcinoma	Anti-PD-1 + D-CIK	Phase 1/2
NCT03867084	Unknown	Hepatocellular Carcinoma	Pembrolizumab	Phase 3
NCT02795429	Active, not recruiting	Hepatocellular Carcinoma	PDR001 (anti-PD-1) + INC280	Phase 1/2
NCT03099564	Recruiting	Hepatocellular Carcinoma	Pembrolizumab	Phase 1
NCT03949231	Active, not recruiting	Hepatocellular Carcinoma	Toripalimab	Phase 3
NCT03062358	Active, not recruiting	Hepatocellular Carcinoma	Pembrolizumab	Phase 3
NCT03474640	Recruiting	Various solid cancers including hepatocellular carcinoma	Toripalimab	Phase 1
NCT02947165	Recruiting	Various solid cancers including hepatocellular carcinoma	PDR001 + NIS793 (anti-TGF)	Phase 1
NCT03836352	Recruiting	Various solid cancers including hepatocellular carcinoma	Pembrolizumab in combination with various drugs	Phase 2
NCT03170960	Recruiting	Various solid cancers including hepatocellular carcinoma	Atezolizumab + Cabozantinib	Phase 1/2
NCT03941873	Recruiting	Various solid cancers including hepatocellular carcinoma	Tislelizumab + Sitravatinib	Phase 1/2
NCT03638141	Recruiting	Hepatocellular Carcinoma	Durvalumab and Tremelimumab	Phase 2
NCT02658019	Active, not recruiting	Hepatocellular Carcinoma	Pembrolizumab	Phase 2
NCT02702414	Active, not recruiting	Hepatocellular Carcinoma	Pembrolizumab	Phase 2
NCT03539822	Recruiting	Various GI cancers including hepatocellular carcinoma	Durvalumab + Cabozantinib	Phase 1
NCT03228667	Recruiting	Various solid cancers including hepatocellular carcinoma	Pembrolizumab, Nivolumab, Atezolizumab, Avelumab, and ALT-803	Phase 2
NCT03713593	Active, not recruiting	Hepatocellular Carcinoma	Pembrolizumab + Lenvatinib	Phase 3
NCT03829501	Recruiting	Various solid cancers including hepatocellular carcinoma	Atezolizumab + KY1044	Phase 1/2
NCT03563170	Active, not recruiting	Hepatocellular Carcinoma	Avelumab in combination with various drugs	Phase 1/2
NCT04246177	Recruiting	Hepatocellular Carcinoma	Pembrolizumab with lenvatinib with TACE	Phase 3
NCT04170556	Recruiting	Hepatocellular Carcinoma	Regorafenib followed by pembrolizumab	Phase 1/2
CTLA-4				
NCT02821754	Recruiting	Liver and biliary tract cancer	Tremelimumab and durvalumab	Phase 2
NCT04430452	Not yet recruiting	Hepatocellular Carcinoma	Radiation followed by durvalumab with or without tremelimumab	
Vaccines				
NCT03674073	Recruiting	Hepatocellular Carcinoma	Neoantigen vaccine	Phase 1
NCT02232490	Recruiting	Hepatocellular Carcinoma	hepcortespenlisimut-L	Phase 3
NCT03086564	Unknown	Hepatocellular Carcinoma	HBV expressing DCs	Phase 1/2
NCT02432963	Active, not recruiting	Various solid cancers including hepatocellular carcinoma	Modified Vaccinia Virus Expressing p53 + Pembrolizumab	Phase 1
NCT04317248	Not yet recruiting	Hepatocellular Carcinoma	Dendritic Cell Vaccine	Phase 2
NCT04251117	Recruiting	HCC	GNOS-PV02 (Peptide Vaccine)	Phase 1/2
NCT04248569	Recruiting	Hepatocellular Carcinoma	Peptide Vaccine	Phase 1
NCT03311334	Recruiting	Various solid cancers including hepatocellular carcinoma	DSP-7888 (Peptide Vaccine)	Phase 1/2
NCT01266707	Unknown status	Hepatocellular Carcinoma	Peptide Vaccine	Phase 1
NCT00610389	Unknown status	Various solid cancers including hepatocellular carcinoma	Dendritic Cell Vaccine	Phase 2
Oncolytic viruses				
NCT02562755	Active, not recruiting	Hepatocellular Carcinoma	Pexastimogene Devacirepvec (Pexa Vec) + Sorafenib	Phase 3
NCT01628640	Active, not recruiting	Various solid cancers including hepatocellular carcinoma	Recombinant VSV-expressing Interferon-beta	Phase 1
NCT03647163	Recruiting	Various solid cancers including hepatocellular carcinoma	VSV-IFNÎ^2^-NIS + Pembrolizumab	Phase 1
NCT03313596	Recruiting	Hepatocellular Carcinoma	ADV-Tk	Phase 3
NCT02293850	Recruiting	Hepatocellular Carcinoma	Telomelysin (adenovirus)	Phase 1
ACT				
NCT03175679	Unknown	Hepatocellular Carcinoma	iNKT cells	Phase 1
NCT03175705	Unknown	Hepatocellular Carcinoma	HCC antigens-specific CD8+ T lymphocytes	Phase 1
NCT03441100	Recruiting	Various solid cancers including hepatocellular carcinoma	IMA202 (TCR-engineered T cells)	Phase 1
NCT03980288	Recruiting	Hepatocellular Carcinoma	CAR-GPC3 T Cells	Phase 1
